# In-Hospital Outcomes in Patients Admitted to the Intensive Care Unit after a Return Visit to the Emergency Department

**DOI:** 10.3390/healthcare9040431

**Published:** 2021-04-07

**Authors:** Chun-Fu Lin, Yi-Syun Huang, Ming-Ta Tsai, Kuan-Han Wu, Chien-Fu Lin, I-Min Chiu

**Affiliations:** 1 Department of Emergency Medicine, Kaohsiung Chang Gung Memorial Hospital, No. 123, Dapi Rd. Niaosong Dist., Kaohsiung 83301, Taiwan; vincetofu@gmail.com (C.-F.L.); neuhaus@cgmh.org.tw (Y.-S.H.); kabadada@gmail.com (M.-T.T.); hayatowu1120@gmail.com (K.-H.W.); u9801314@cmu.edu.tw (C.-F.L.); 2 Department of Computer Science and Engineering, National Sun Yet-Sen University, Kaohsiung 804, Taiwan

**Keywords:** outcomes, intensive care unit, return visit, emergency department

## Abstract

Background: Intensive care unit (ICU) admission following a short-term emergency department (ED) revisit has been considered a particularly undesirable outcome among return-visit patients, although their in-hospital prognosis has not been discussed. We aimed to compare clinical outcomes between adult patients admitted to the ICU after unscheduled ED revisits and those admitted during index ED visits. Method: This retrospective study was conducted at two tertiary medical centers in Taiwan from 1 January 2016 to 31 December 2017. All adult non-trauma patients admitted to the ICU directly via the ED during the study period were included and divided into two comparison groups: patients admitted to the ICU during index ED visits and those admitted to the ICU during return ED visits. The outcomes of interest included in-hospital mortality, mechanical ventilation (MV) support, profound shock, hospital length of stay (HLOS), and total medical cost. Results: Altogether, 12,075 patients with a mean (standard deviation) age of 64.6 (15.7) years were included. Among these, 5.3% were admitted to the ICU following a return ED visit within 14 days and 3.1% were admitted following a return ED visit within 7 days. After adjusting for confounding factors for multivariate regression analysis, ICU admission following an ED revisit within 14 days was not associated with an increased mortality rate (adjusted odds ratio (aOR): 1.08, 95% confidence interval (CI): 0.89 to 1.32), MV support (aOR: 1.06, 95% CI: 0.89 to 1.26), profound shock (aOR: 0.99, 95% CI: 0.84 to 1.18), prolonged HLOS (difference: 0.04 days, 95% CI: −1.02 to 1.09), and increased total medical cost (difference: USD 361, 95% CI: −303 to 1025). Similar results were observed after the regression analysis in patients that had a 7-day return visit. Conclusion: ICU admission following a return ED visit was not associated with major in-hospital outcomes including mortality, MV support, shock, increased HLOS, or medical cost. Although ICU admissions following ED revisits are considered serious adverse events, they may not indicate poor prognosis in ED practice.

## 1. Introduction

In recent decades, unscheduled return visits to emergency departments (EDs), usually defined as return visits soon after the last ED discharge, were considered important quality indicators of ED care and were routinely monitored in clinical practice [1,2,3]. Hospital admission after an ED revisit was often due to rapid deterioration after ED discharge or a serious adverse event [4,5,6]. Recently, this concept has been challenged by several studies [7,8,9]. A study conducted in 2015 revealed that ED return visits exhibited 66% sensitivity and 68% specificity in the identification of quality-related returns [10]. Moreover, a multicenter study showed that patients admitted during a return ED visit exhibited lower in-hospital mortality, intensive care unit (ICU) admission rates, and in-hospital costs when compared with patients who were hospitalized during initial ED visits [11].

ICU admission following a short-term ED revisit is an uncommon event and has been considered a particularly undesirable outcome among return-visit patients, although their in-hospital prognosis has not been discussed extensively. A previous study reported that approximately 13.9% of the patients admitted to the ICU during a return visit had deviation in care during the initial ED visit [12]. Another study identified several prognostic factors for poor outcomes in patients admitted to the ICU following a return visit. These included older age, multiple comorbidities, and worsening severity index [13]. However, we observed that these prognostic factors were common among all ICU-admitted patients in previous studies [14,15,16].

A recent study demonstrated that there was no increased risk of mortality or increased hospital length of stay (HLOS) in children admitted to the ICU after unscheduled ED revisits when compared with those admitted during initial ED visits [17]. However, to the best of our knowledge, no study has directly compared the clinical outcomes between adult patients admitted to the ICU following return ED visits and those admitted to the ICU during initial ED visits. In this study, we aimed to analyze and compare the in-hospital outcomes and hypothesized that patients that had ICU admission following a revisit to the ED would exhibit poorer outcomes than patients admitted during the initial ED visit.

## 2. Materials and Methods

### 2.1. Patient Population

This retrospective study was conducted at two tertiary medical centers in Taiwan from 1 January 2016 to 31 December 2017. One of the hospitals was located in northern Taiwan and had approximately 130,000 annual ED visits. The other was in southern Taiwan and had approximately 110,000 annual ED visits. Both the hospitals were the largest medical centers in their respective metropolitan areas. The study protocol was approved by the institutional review board of both the hospitals. The study was conducted according to the guidelines of the Declaration of Helsinki, and approved by the Institutional Review Board of Chang Gung Medical Foundation (protocol code 202002043B0 and date of approval 1 December 2020).

All adult non-trauma patients admitted to the ICU directly via the ED during the study period were included. Patients discharged against medical advice, patients transferred to another hospital after admission, and patients who had a history of hospital admission within 14 days of the ED visit were excluded.

All included patients were divided into two comparison groups: patients admitted to the ICU during an index ED visit and those admitted to the ICU during a return ED visit. An index ED visit was defined as the first ED visit for a unique patient visit with no prior visit during the past 14 days. A recent study that determined the time of an ED revisit suggested that 9 days was the most reasonable cutoff for identification of acute ED revisits [18]. To include most of the return visits, we classified a return ED visit into two separate classes: a return visit within 14 days and a return visit within 7 days from the last ED discharge. Patients with an irrelevant diagnosis between the initial and the return visits were excluded before further analysis.

Patient demographics and clinical characteristics including age, sex, comorbidities, initial vital signs at the ED, severity index upon ICU admission, and diagnosis at ICU admission were collected for comparison. Two severity indices were evaluated: Acute Physiology and Chronic Health Evaluation (APACHE) score [19] and Simplified Acute Physiology Score (SAPS) [20]. Comorbidities were recorded and diagnoses at admission were classified based on the International Classification of Diseases (tenth edition). All data were directly retrieved from the hospital’s research database and the information was de-identified before further analysis.

### 2.2. Outcome Measures and Statistical Analysis

The outcome measures of interest included in-hospital mortality, having undergone surgery, mechanical ventilation (MV) support, profound shock that required inotropic agents, HLOS, and total medical cost. Patients who died in the ED after the return visit and before the ICU admission were included under in-hospital mortality. HLOS was analyzed after excluding patients who died during admission and the medical costs were converted to US dollars (USD).

Data are presented as mean (standard deviation (SD)) for continuous variables, proportions for nominal variables, and median (interquartile range) for ordinal variables. We performed a Student’s *t*-test and two-tailed chi squared analysis to determine the parameters that correlated with ICU admission following an index ED visit and a return ED visit. To evaluate the adjusted differences in outcomes between the groups, multivariable regression models were developed controlling for age, sex, and comorbidities. Statistical significance was set at *p* ≤ 0.05. Statistical analyses were performed using IBM SPSS Statistics for Mac, version 26 (IBM Corp., Armonk, NY, USA).

## 3. Results

During the study period, 634,454 patients visited the study hospitals. Among these, 12,075 patients met the inclusion criteria. The mean age (SD) of the included patients was 64.6 (15.7) years and 4072 (33.7%) were women. Altogether, 5.3% of the patients were admitted to the ICU after an ED revisit within 14 days and 3.1% of patients were admitted after an ED revisit within 7 days.

When compared with patients admitted to the ICU during an index visit, those admitted to the ICU after a 14-day return visit were more likely to exhibit tachycardia (*p* = 0.007), hypotension (systolic as well as diastolic, *p* < 0.001 for both), and hypoxia (*p* = 0.003) in the ED. Patients admitted following a 7-day return visit also demonstrated similar findings when compared with those admitted to the ICU following an index ED visit, except initial oxygen saturation (no significant difference, *p* > 0.05) (Table 1).

Patients that had ICU admission after 14-day and 7-day ED visits exhibited a higher incidence of end-stage renal disease (ESRD) (15.6% and 14.8%, respectively, vs. 8.0%, *p* < 0.001), liver cirrhosis (12.9% and 12.0%, respectively, vs. 8.1%, *p* = 0.001), and malignancy (22.1% and 24.8%, respectively, vs. 12.4%, *p* < 0.001) compared to patients admitted during an index visit.

A lower percentage of acute coronary syndrome was noted among both 14-day (*p* < 0.001) and 7-day (*p* = 0.006) return visit groups compared to patients that had ICU admission during an index visit. Other diagnoses at ICU admission showed no significant differences.

Two severity indices (APACHE score and SAPS) were documented in this study. There were no significant differences in these scores among the studied groups. Furthermore, neither the 14-day return visit group nor the 7-day return visit group exhibited significant differences in in-hospital outcomes including mortality, having undergone surgery, MV support, shock, HLOS, and total medical cost when compared with patients admitted during an index ED visit (Table 1).

After adjusting for confounding factors for the multivariate regression analysis, ICU admission following a 14-day ED visit was not associated with an increased mortality rate (adjusted odds ratio (aOR): 1.08, 95% confidence interval (CI): 0.89 to 1.32), having undergone surgery (aOR:0.93, 95% CI: 0.74 to 1.11), MV support (aOR:1.06, 95% CI: 0.89 to 1.26), profound shock (aOR: 0.99, 95% CI: 0.84 to 1.18), prolonged HLOS (difference: 0.04 days, 95% CI: −1.02 to 1.09), and increased total medical cost (difference: USD 361, 95% CI: −303 to 1025). Similar results were observed after the regression analysis for patients that had a 7-day return visit (Table 2).

## 4. Discussion

Numerous studies have discussed the predictive and prognostic factors of ED revisit in recent decades. To the best of our knowledge, this is the first study to compare the in-hospital outcomes between adult patients admitted to the ICU following a return ED visit and those admitted during an index ED visit. Our findings opposed the hypothesis that ICU admission after ED revisit was associated with worse clinical outcomes. The results of both 14-day and 7-day return visit groups were consistent.

Previous studies have shown that patients revisiting the ED had fewer comorbidities and lower triage acuity [11,21]. Our study demonstrated different findings among patients who revisited the ED and were subsequently admitted to the ICU. We observed that patients that had ICU admission following 14-day and 7-day return ED visits were more likely to be exhibit hypotension, tachycardia, and hypoxia in the ED compared to patients admitted during an index ED visit. The differences, though trivial, were statistically significant as clinical findings. These findings may be explained by the fact that a considerable number of patients were admitted to the ICU during an index visit due to acute coronary syndrome, which usually does not present with hypotension and tachycardia.

Patients admitted to the ICU during a return visit exhibited a higher incidence of ESRD, liver cirrhosis, and malignancy. This finding is consistent with the results of previous studies, which suggested that patients who revisited the ED with underlying diseases such as liver cirrhosis and cancer were more likely to have adverse outcomes including ICU admission and death after the return visit [6,22]. Patients with underlying comorbidities may have a greater tendency for disease progression and can deteriorate quickly within several days.

There were no significant differences in patients’ severity index (APACHE score and SAPS) between the study groups. Compared to general ward admission, decisions regarding ICU admission might be more objective and the decision was usually made based on certain criteria such as mechanical ventilation support required, profound shock, or a specific disease requiring intensive monitoring, including severe stroke and acute myocardial infarction.

In this study, the major prognostic outcomes under focus were in-hospital mortality, HLOS, MV support, shock, and total medical cost. Patients that had 14-day and 7-day return ED visits who were subsequently admitted to the ICU did not exhibit significant differences in these primary outcomes (Table 2). Similar results were observed in a previous study in pediatric patients [9,17]. A study that examined in-hospital clinical outcomes among patients hospitalized during an unscheduled return visit to the ED revealed significantly lower mortality, lower HLOS, and lower hospital cost in patients that had unscheduled ED revisits [11]. It seems that unscheduled ED revisits did not have an association with poorer outcomes among patients admitted to the ICU or to the general ward. Similar categorical distributions of diagnoses at admission between patients admitted during an index visit and those admitted during a return visit support this point of view.

Few studies have discussed the outcomes of patients that had delayed ICU admission after a visit to the ED and the delay was attributed to the waiting time in the ED [23,24,25]. Although the results varied, most of the studies agreed that delayed ICU admission was associated with increased HLOS and mortality, which was contrary to the findings from our study. The findings may not be comparable, since patients admitted to the ICU during a return visit did not meet the criteria for ICU admission on their index visit.

The present study has several limitations. First, it was a retrospective study and the data were collected from two medical centers belonging to the same medical foundation. This may limit the generalizability of the results. Second, we could not follow and include patients who visited other hospitals on their return visit. This may have underestimated the number of patients that had return visits. However, the two studied hospitals were the largest medical centers in their respective neighborhoods and ICU admission is one of the most critical conditions, which is usually handled at tertiary centers. Hence, it is reasonable to assume that the possibility of losing track of patients was low. Third, the study was performed some time before the recent global health event. The study result may not precisely apply to healthcare facilities that have been deeply affected by the global pandemic.

## 5. Conclusions

ICU admission following a return ED visit was not associated with major in-hospital outcomes, including mortality, MV support, shock, and increased HLOS. Moreover, it did not result in increased medical costs during admission. These findings suggest that although ICU admission associated with return ED visits was considered a serious adverse event in previous studies, it may not indicate poor prognosis in ED practice.

## Figures and Tables

**Table 1:** Demographics, clinical characteristics, and in-hospital outcomes of patients admitted to the ICU following an index ED visit, 14-day return ED visit, and 7-day return ED visit.

	Index ED Visit	14-Day Return ED Visit	*p*-Value	7-Day Return ED Visit	*p*-Value
	*n* = 11,434	*n* = 641		*n* = 371	
Age, mean (SD)	64.6 (15.9)	65.6 (15.4)	0.094	64.5 (14.9)	0.931
Female sex, %	35.7	36.4	0.785	36.4	0.785
Initial vital signs					
Temperature, mean (SD)	36.6 (1.6)	36.5 (2.7)	0.261	36.6 (2.2)	0.777
Heart rate, mean (SD)	95 (27.9)	98 (28.7)	0.007	98 (27.6)	0.045
Systolic BP, mean (SD)	140 (42.4)	133 (41.2)	<0.001	134 (40.8)	0.013
Diastolic BP, mean (SD)	82 (24.6)	78 (23.2)	<0.001	79 (22.8)	0.029
O_2_ saturation %, mean (SD)	92 (14.1)	89 (17.6)	0.003	90 (16.3)	0.053
Comorbidities					
Diabetes mellitus, %	22.6	22.2	0.805	21.8	0.785
Heart failure, %	14.2	15.6	0.269	13.7	0.874
ESRD, %	8.0	14.8	<0.001	15.6	<0.001
Liver cirrhosis, %	8.1	12.0	0.001	12.9	0.001
Malignancy, %	12.4	24.8	<0.001	22.1	<0.001
Severity index					
APACHE score, median (IQR)	18 (13–24)	18 (14–25)	0.102	17 (13–25)	0.897
SAPS, median (IQR)	38 (29–50)	39 (31–50)	0.672	39 (30–47)	0.332
Diagnosis at admission					
Acute coronary syndrome, %	21.7	15.8	<0.001	16.2	0.006
Acute stroke, %	19.8	20.5	0.617	20.5	0.974
Respiratory failure, %	12.5	11.0	0.342	10.8	0.245
Sepsis, %	7.4	7.5	0.961	7.8	0.822
Outcome					
Death, %	19.3	21.0	0.425	20.4	0.474
Undergone surgery, %	11.3	10.7	0.784	10.9	0.833
MV support, %	62.5	63.6	0.702	66.1	0.054
Shock, %	30.7	32.9	0.392	33.2	0.172
HLOS in days, median (IQR)	12 (6–22)	12 (6–22)	0.960	14 (8–23)	0.353
Medical cost in US dollars, median (IQR)	3191 (6010–10,812)	3164 (6096–10,867)	0.300	3581 (6507–10,891)	0.128

ICU, intensive care unit; ED, emergency department; SD, standard deviation; BP, blood pressure; ESRD, end-stage renal disease; APACHE, Acute Physiology and Chronic Health Evaluation; SAPS, Simplified Acute Physiology Score; IQR, interquartile range; MV, mechanical ventilation; HLOS, hospital length of stay.

**Table 2:** Regression analysis of ICU admission following return ED visit with respect to in-hospital outcome and medical cost.

	14-Day Return Visit		7-Days Return Visit	
	aOR/Difference (95% CI)	*p*-Value	aOR/Difference (95% CI)	*p*-Value
Mortality	1.08 (0.89 to 1.32)	0.438	1.12 (0.87 to 1.44)	0.387
Undergone surgery	0.93 (0.74 to 1.11)	0.564	0.96 (0.77 to 1.15)	0.717
MV support	1.06 (0.89 to 1.26)	0.505	0.96 (0.77 to 1.19)	0.709
Shock	0.99 (0.84 to 1.18)	0.937	0.99 (0.80 to 1.24)	0.995
HLOS	0.04 (−1.02 to 1.09)	0.947	0.19 (−1.18 to 1.57)	0.783
Medical cost	361 (−303 to 1025)	0.287	379 (−483 to 1240)	0.389

ICU, intensive care unit; ED, emergency department; MV, mechanical ventilation; HLOS, hospital length of stay; aOR, adjusted odds ratio; CI, confidence interval.

## Data Availability

Restrictions apply to the availability of these data. Data was obtained from Chang Gung Medical Foundation and are available from the corresponding author with the permission of Chang Gung Medical Foundation.
